# Sodium Retention in Large Herbivores: Physiological Insights and Zoogeochemical Consequences

**DOI:** 10.1002/jez.2924

**Published:** 2025-04-17

**Authors:** Andrew J. Abraham, Ethan S. Duvall, Christopher E. Doughty, Barbara Riond, Sylvia Ortmann, Melissa Terranova, Elizabeth le Roux, Marcus Clauss

**Affiliations:** ^1^ Section of EcoInformatics and Biodiversity, Department of Biology Centre for Ecological Dynamics in a Novel Biosphere (ECONOVO) Aarhus University Aarhus Denmark; ^2^ School of Informatics, Computing and Cyber Systems Northern Arizona University Flagstaff Arizona USA; ^3^ Department of Ecology and Evolutionary Biology Cornell University Ithaca New York USA; ^4^ Clinical Laboratory, Department for Clinical Diagnosis and Services Vetsuisse Faculty University of Zurich Zurich Switzerland; ^5^ Leibniz‐Institute for Zoo and Wildlife Research (IZW) Berlin Germany; ^6^ AgroVet‐Strickhof, ETH Zurich Lindau Switzerland; ^7^ Mammal Research Institute, Faculty of Natural and Agricultural Sciences University of Pretoria Pretoria South Africa; ^8^ Clinic for Zoo Animals, Exotic Pets and Wildlife, Vetsuisse Faculty University of Zurich Zurich Switzerland; ^9^ AgroVet‐Strickhof, Vetsuisse Faculty University of Zurich Lindau Switzerland

**Keywords:** dispersal, feces, hypertension, passage time, sodium, urine

## Abstract

The assimilation, retention, and release of nutrients by animals fundamentally shapes their physiology and contributions to ecological processes (e.g., zoogeochemistry). Yet, information on the transit of nutrients through the bodies of large mammals remains scarce. Here, we examined how sodium (Na), a key element for animal health and ecosystem functioning, travels differently through fecal and urinary systems of cows (*Bos taurus*) and horses (*Equus ferus caballus*). We provided a large dose of Na and compared its timing of release in feces and urine to that of nonabsorbable markers. Na excretion by urine occurred approximately twice as fast as excretion by feces, yet both were shorter than indigestible particle markers. These differences correspond to rapid absorption of Na in the upper gastrointestinal tract and transport by blood to the kidneys (urine Na excretion) or resecretion of Na into the lower intestinal tract (fecal Na excretion). Interestingly, for cows, we found a second peak of Na excretion in urine and feces > 96 h after dosage. This result may indicate that surplus Na can be rapidly absorbed and stored in specific body cells (e.g., skin), from which it is later released. Using a propagule dispersal model, we found that the distance of cattle‐ and horse‐driven nutrient dispersal by urine was 31% and 36% less than the fecal pathway and 60% and 41% less than the particle marker pathway, which is commonly used to estimate nutrient dispersal. Future physiological and zoogeochemical studies should resolve different pathways of nutrient retention and release from large mammals.

## Summary

- Transit of sodium through an animals body is twice as fast in urine than feces.
- The magnitude of nutrient redistribution by large herbivores is constrained by specific pathways of element assimilation, retention and release.

## Graphical abstract

Sodium travels twice as fast through the bodies of large herbivores in urine than feces. The specific pathway of element assimilation, retention and release exerts an important constraint on the contributions of wildlife and domestic livestock to nutrient recycling and redistribution.

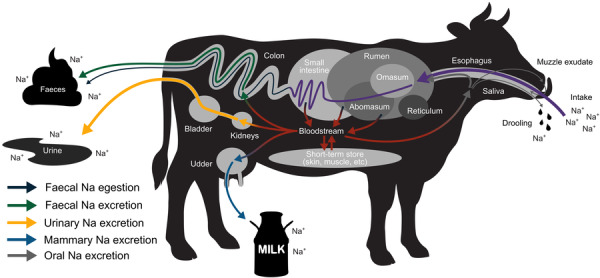

## 1 Introduction

The assimilation, retention, and release of nutrients by animals underpins multiple aspects of their biology and ecology (Robbins 2012; Subalusky and Post 2019). For example, an animals’ nutritional requirements, which dictate various behaviors (e.g., diet, movement patterns) and life‐history traits (e.g., reproductive cycles), are directly related to the assimilation efficiency and retention time of nutrients within their bodies (Birnie‐Gauvin et al. 2017). This is well demonstrated in large mammalian herbivores, which are considered disproportionately important for biosphere functioning (Enquist et al. 2020). For instance, herbivores that contain a hindgut fermenting digestive system often must consume more forage than an equally sized ruminant to compensate for their lower assimilation efficiency (Clauss et al. 2015; Müller et al. 2013). These differences can have important implications for large herbivore survival, as well as their contributions to ecosystems via top‐down (e.g., herbivory) and bottom‐up (e.g., nutrient recycling) processes (Pringle et al. 2023).

Among the 25+ elements required for life (Kaspari and Powers 2016), sodium (Na) is recognized for its critical role in shaping animal physiology and ecology (Kaspari 2020). This is particularly true for large herbivores, which may be especially vulnerable to Na deficiency in Na‐poor environments (Duvall et al. 2023). Na deficiency risks failure of metabolic, neural, and muscular processes, yet excess Na intake can also lead to harmful effects (e.g., hypertension) (Robbins 2012). Thus, animals aim to keep bodily Na concentrations within a narrow osmotic window (Grace et al. 1979; Robbins 2012). To facilitate this, many animals—particularly plant‐consuming herbivores whose natural diet is often Na‐depleted—have evolved a strong taste for Na, which helps them balance Na intake (Taruno and Gordon 2023). Additionally, animals often display remarkable Na conservation in low‐Na environments with minimal amounts lost in excreta (e.g., via very low urine Na concentrations) (Hellgren and Pitts 1997). By contrast, in high‐Na environments, animals regulate excess Na from their bodies via excreta and may also increase water intake to balance fluid osmolarity in cells (Bernal et al. 2023; Jansson and Dahlborn 1999). Despite the importance of these physiological controls on Na balance (Hellgren and Pitts 1997), differences in the retention time of Na through different pathways in the bodies of large herbivores have received little attention.

When an animal consumes nutrients, a portion is assimilated via the digestive tract with the remainder ejected in feces (egestion). Of the proportion assimilated, some becomes integrated into body tissues while the rest is excreted from the body as waste products (excretion). Na has a very high assimilation rate (> 90%) and excretion primarily occurs via the renal system (urine), but some Na may be resecreted back into the large intestine and regulated in feces (Grace et al. 1979). Additional Na may be lost from the body through smaller or sporadic excretion pathways, such as saliva drooling, lactation, skin sloughing, feather molting or antler shedding (Subalusky and Post 2019). For example, Hellgren and Pitts (1997) found that for white‐tailed deer (*Odocoileus virginianus*) Na is primarily excreted in feces under low‐Na intake but in urine when Na is in excess, yet Na sequestered in antlers was 1–2 orders of magnitude smaller. While such research has examined the relative magnitude of Na losses via different egestion and excretion pathways, differences in the retention time Na transits an organism's body between pathways have not yet been explored. Evaluating these temporal differences may be crucial for a deeper understanding of the physiological mechanisms involved in Na regulation, as well as quantifying animal roles in ecosystem Na cycling.

Indeed, research has increasingly highlighted the important roles of animals in nutrient cycling and redistribution (Abraham et al. 2021; Doughty et al. 2016; Trepel et al. 2024), including the potential importance of large herbivores in Na poor environments (Doughty et al. 2016). For example, the location, quantity, and form of Na ingestion and release by animals may influence the rate of key ecological processes (e.g., microbial decomposition) and patterns of biodiversity (e.g., soil fauna abundance and composition) (Kaspari 2020). Existing zoogeochemical models, however, tend to amalgamate multiple egestion and excretion pathways into a single flux, characterizing key functions, such as nutrient passage time, by the fecal egestion pathway, for which most information is available (e.g., Abraham et al. 2021; Doughty et al. 2016). Yet, as Na is almost entirely absorbed into the bloodstream (Hellgren and Pitts 1997), deviations in the retention time by different excretion pathways (i.e., urinary Na or resecreted fecal Na) may have appreciable impacts on nutrient dispersal distances. Improving the parameterization of zoogeochemical models is considered necessary for guiding strategies of ecological restoration, Earth system stewardship, and climate change adaptation (Abraham et al. 2023; Abraham et al. 2022; Malhi et al. 2022).

In the present study, we undertook experimental feeding trials to compare temporal differences in Na transit between the two major nutrient release pathways in large mammalian herbivores: the fecal and urinary systems. Notably, the fecal pathway consists of the outcome of two different mechanisms: egestion, which represents Na in resdiues of food that was not absorbed in the digestive tract, and excretion, which represents components that were absorbed in the upper digestive tract but are resecreted back into the lower tract. We examined two large herbivore species: cattle (*Bos taurus*; ruminant) and horses (*Equus ferus caballus*; hindgut fermenter). We then integrated information from our feeding trials into a propagule transport model to explore how differences in retention time between digestive and urinary pathways influence a key zoogeochemical process: lateral nutrient dispersal.

## 2 Materials and Methods

This experiment was conducted at the AgroVet‐Strickhof research facility, Switzerland, during February 2024, under the experimental licence 35775 | ZH059/2023.

### 2.1 Study Animals and Husbandry

We used four rumen‐fistulated Original Brown Swiss cows (body mass range: 700–740 kg) in the final stage of lactation (milk yield 6–8 L/d) (see Table 1 for information on individual animals). Animals were part of the AgroVet‐Strickhof research herd living in a free stall, and had received rumen fistulae several years before the present study. They were brought to the tie stall (to which they were habituated from other previous experiments) 3 days before the experiment. Each cow was tethered in an individual stall with chopped straw bedding and ad libitum access to water. The rear end of the stand was formed by a grid through which defecated feces fell into a waste canal; sufficient material remained on these grids at each defecation for sampling. Water consumption was electronically recorded every 15 min in units of 1000 mL. Cows were kept on total mixed ration based on grass and maize silage for the duration of the experiment, which had a Na concentration of 5185 mg kg^−1^ dry matter. Each morning, 30 kg of fresh total mixed ration was provided onto a feeding platform, with an additional 20 kg supplied in the evening. At the beginning of each day, all remaining food was collected and weighed. As there were always leftovers, this was considered feeding for ad libitum consumption. One day before the experiment, cows were fitted with urinals custom‐made from diving suits attached around the vulva of the cows and fixed by hook‐and‐loop fastener straps glued (Ergo 5011; Kisling AG, Wetzikon, Switzerland) onto the skin. The urinals were connected through a pipe to a canister on the ground for urine collection. Feces were removed regularly from behind the animals.

**TABLE 1:** Animals used in this study and the mean retention time (MRT) for passage markers and sodium (Na) following a pulse dosage of salt (NaCl).

Species	Name	Age (years)	Mass (kg)	Passage marker mean retention time (MRT) (h)			Sodium (Na) mean retention time (h)		
				Co‐ETDA solute	2 mm particle	10 mm particle	Urine Na	Urine Na: creatine	Fecal Na
Cow	Leila	6	740	27	50	54	16	13	32
Cow	Luna	6	740	22	41	48	13	11	27
Cow	Rubi	4	720	22	43	49	16	14	27
Cow	Herta	6	700	25	42	51	17	16	26
Mean ± SD				24.1 ± 2.6	44.2 ± 4.2	50.4 ± 2.5	15.5 ± 1.4	13.6 ± 1.9	28.0 ± 2.6
Horse	Mixxa	23	520	29	30	30	19	18	28
Horse	Victoire	14	520	26	29	29	NA	NA	32
Horse	Chilli	6	550	36	38	38	17	17	28
Horse	Rattma	18	500	27	31	31	15	14	29
Mean ± SD				29.7 ± 4.1	32.0 ± 4.4	32.1 ± 4.3	17.1 ± 1.6	16.5 ± 1.9	29.5 ± 2.0

Four female warmblood horses (body mass range: 500–550 kg) were kept together in their usual enclosure, which provided access to covered inside and an outdoor area (see Table 1 for information on individual animals). All animals had ad libitum access to water from a common drinking station; water consumption could not be measured. The usually available salt lick was removed from the enclosure 6 weeks before the start of our experiment. During these 6 weeks, the horses were habituated every 3–4 days to receiving ~1 kg of dry mash (3777 mg kg^−1^ dry matter) per horse into which various amounts of salt (NaCl) was mixed. It was determined in these pilot experiments that maximum voluntary intake would be about 150 g NaCl in this mixture. We stopped pilot experiments 2 weeks before the experiment. Hay was provided ad libitum throughout the preparation stage and the experiment, which had a Na concentration of 196 mg kg^−1^ dry matter. An additional source of straw was also available with unknown sodium concentration. In this setup, food intake could not be measured.

### 2.2 Passage Experiment

We first collected control feces and urine from all animals (*n* = 2–3 per individual) on the day before the salt and passage marker dosage treatment. For cows, samples of feces were collected from the defecation grid, and samples of urine were taken from the canister. For horses, continuous observation was necessary to be able to ascribe feces to an individual. This was achieved by two persons (AJA and MC) taking turns in 4‐h shifts. Urine was collected from horses using a container attached to a pole. Whereas three horses tolerated urine collection, one horse tried to avoid this, and we suspected that it must have sometimes urinated while the observer was distracted by picking up feces or sampling urine of other individuals; this individual was therefore excluded from horse averages for urine measures, but still included for fecal measurements.

For each animal, we then administered a pulse dose of salt (NaCl; 400 and 150 g per cow and horse, respectively). We used a large pulse of NaCl to maximize the opportunity of observing a signal in retention times across both urinary and fecal pathways. However, we ensured to administer within safe Na limits. According to veterinary sources (CliniTox https://www.vetpharm.uzh.ch/clinitox/toxdb/WDK_072.htm and (Coenen and Vervuert 2019), clinically dangerous doses in animals with ad libitum water access would have been 1.2 kg of salt for cows and 3 kg salt for horses. In parallel, we administered three commonly‐used markers for measuring digesta retention: (i) Co‐ethylenediaminetetraacetic acid (Co‐EDTA; solute marker representing the passage of fluid; 7 and 5 g per cow and horse, respectively), (ii) Cr‐mordanted fiber (2 mm particle marker; 65 and 50 g) and (iii) Ce‐mordanted fiber (10 mm particle marker; 65 and 50 g). All markers were prepared following Udén et al. (1980). In cows, salt and markers were manually inserted into the top particle layer of the rumen contents via the fistula. For horses, the salt and markers were mixed into a portion of 1.5 kg mash and offered for voluntary ingestion. Whereas all horses ingested the majority of this mixture, completeness of ingestion varied, so that the exact ingested dose was unknown. In the case of horses, the salt dose was based on our pilot experiment on voluntary ingestion; in the case of cows, a higher (yet safe) dose was used to achieve a more distinct signal.

For cows, we then collected feces and urine for each individual over 7 days (168 h) at progressively longer intervals (interval on days 1–2 = 4 h; days 3–4 = 6 h; day 5 = 8 h; days 6–7 = 12 h; total *n* = 26 per individual). The defecation grids were checked at least every 4 h to collect feces, even when the respective sampling interval was longer. In this setup, urine and fecal samples were available for all animals at all sampling intervals. At the end of each interval, feces collected during a collection period were mixed homogenously, and a representative sample of about 250 g fresh material was taken. Urine collected in the canisters was weighed before sampling and then ~150 mL extracted via pipette. The canister was cleaned and replaced between each sampling interval. For horses, we only collected feces and urine for 4 days (96 h) due to their shorter gut passage time (Abraham et al. 2021). While every single defecation was collected, these were combined into the same interval samples as for cows during days 1–4 (*n* = 19 per individual). In horses, urine was collected at every observed event and the time recorded. As horse urine contains high levels of calcium (Caple et al. 1982), all horse urine samples were immediately centrifuged for 5 min at 390 × g to prevent analytical interference due to mucus and crystalluria. Feces from all cows and horses were dried at 105°C for 72 h and ground in a feed mill (Schmersal GmbH, Wuppertal, Germany) using a 1 mm matrix. All urine samples were frozen at −20°C.

### 2.3 Sample Analysis

We measured the concentration of Na and passage markers (Co, Cr, Ce) in feces using inductively coupled plasma optical emission spectrometry (ICP‐OES, model Optima 8000, Perkin Elmer) after wet ashing (Frei et al. 2015). For urine, we measured the concentration of Na and creatinine. Urinary element concentrations are susceptible to dilution; for example, in response to the high salt dose, animals may increase drinking water intake and thus their urine volume (Bankir et al. 2017). Creatinine can be used to correct for this dilution effect as the daily excretion of urinary creatinine is considered constant, and hence deviations in its concentration are indication of the degree of dilution (Chizzotti et al. 2008; Rodrigues et al. 2023). Na in urine was measured using indirect ISE (ion‐selective‐electrode) and creatinine was determined using the Jaffé‐method; both analytes were measured using a fully automated chemistry analyzer (Cobas C 501, Roche Diagnostics, Switzerland).

All concentrations of Na, Co, Cr and Ce in feces, and Na and the Na:Creatinine ratio in urine, are reported relative to the baseline values measured before the experiment. For visualization, data were expressed as % of the peak concentration (Matsuda et al. 2015), where positive and negative values indicate increased and decreased concentrations relative to baseline measurements, respectively. To describe the passage of these substances through the animal, the mean retention time was calculated according to Thielemans et al. (1978) as:

(1) $$\mathrm{MRT}=\frac{\sum t_{i}C_{i}{\mathrm{dt}}_{i}}{\sum C_{i}{\mathrm{dt}}_{i}}$$

with *C*_*i*_ = marker concentration in the samples from the interval represented by time *t*_*i*_ (hours after marker administration, using the midpoint of the sampling interval), and *dt*_*i*_ = the interval (h) of the respective sample:

(2) $${\mathrm{dt}}_{i}=\frac{\left(t_{i+1}-t_{i}\right)+(t_{i}-t_{i-1})}{2}$$

It should be noted that the process of Na passage through the organism is different compared to classic passage markers like Co, Cr and Ce; the latter are generally not absorbed, but remain in the gastrointestinal tract. Na, by contrast, is almost completely absorbed (Hellgren and Pitts 1997) and subsequently excreted again into the intestinal tract or into urine. Hence, even if the calculated MRT of Na would yield a similar result as that of Co, Cr or Ce, this would not indicate that these elements move in parallel through the body, just that their respective, different passage routes take a similar amount of time. In cows, a second Na excretion peak was evident after fecal and urinary Na had already returned to baseline. The Na MRT values were only calculated for the time until the initial return to basline.

### 2.4 Quantifying Na Redistribution by Large Herbivores

Difference in the time Na is egested and excreted from an animal's body may influence the magnitude and endpoints of nutrient dispersal by large herbivorous mammals—an important zoogeochemical process (Doughty et al. 2016). To quantify such differences, we modified the propagule dispersal model of Pires et al. (2018). This model was designed to mechanistically encompass seed ingestion, gut passage time, animal movement, and seed deposition to understand how different‐sized animals disperse seeds. However, it is also applicable for quantifying lateral Na dispersal if we consider each “seed” to be a small package of nutrients. We parameterized the model with three different ways of characterizing Na transit through the body: (i) particle markers, (ii) fecal Na and (iii) urinary Na. Particle markers have previously been used to estimate the passage time of nutrients through animal bodies (Abraham et al. 2021; Doughty et al. 2016), but may be inappropriate for Na as this pathway most closely represents the egestion pathway, whereas Na is primarily excreted in urine or resecreted into feces.

Gut passage time in the Pires et al. (2018) model was fit using a single peak gamma distribution, which is described by the rate (MRT) and shape (variance) parameters (Guttal et al. 2011). We therefore first fit a gamma distribution to our fecal and urine passage time data for cows and horses (see section 2.3), setting the rate parameter as the average MRT across individuals in each species (Table 1), and estimating the variance parameter based on a visual assessment of the closest model fit to our empirical concentration curves. For cattle, where we observed two peaks, we calculated retention time until the initial return to baseline as we suspected that the second peak is related to the magnitude of salt administered (see Discussion). All other model parameters were kept identical between simulations. We then ran simulations for a 725 kg cow and a 525 kg horse each to quantify differences in lateral Na dispersal via (i) fecal and (ii) urinary pathways. We generated dispersal kernels from 1000 simulations and calculated the median and interquartile range (25th–75th percentiles).

## 3 Results

### 3.1 Sodium Passage Through the Bodies of Large Herbivores

**FIGURE 1:**
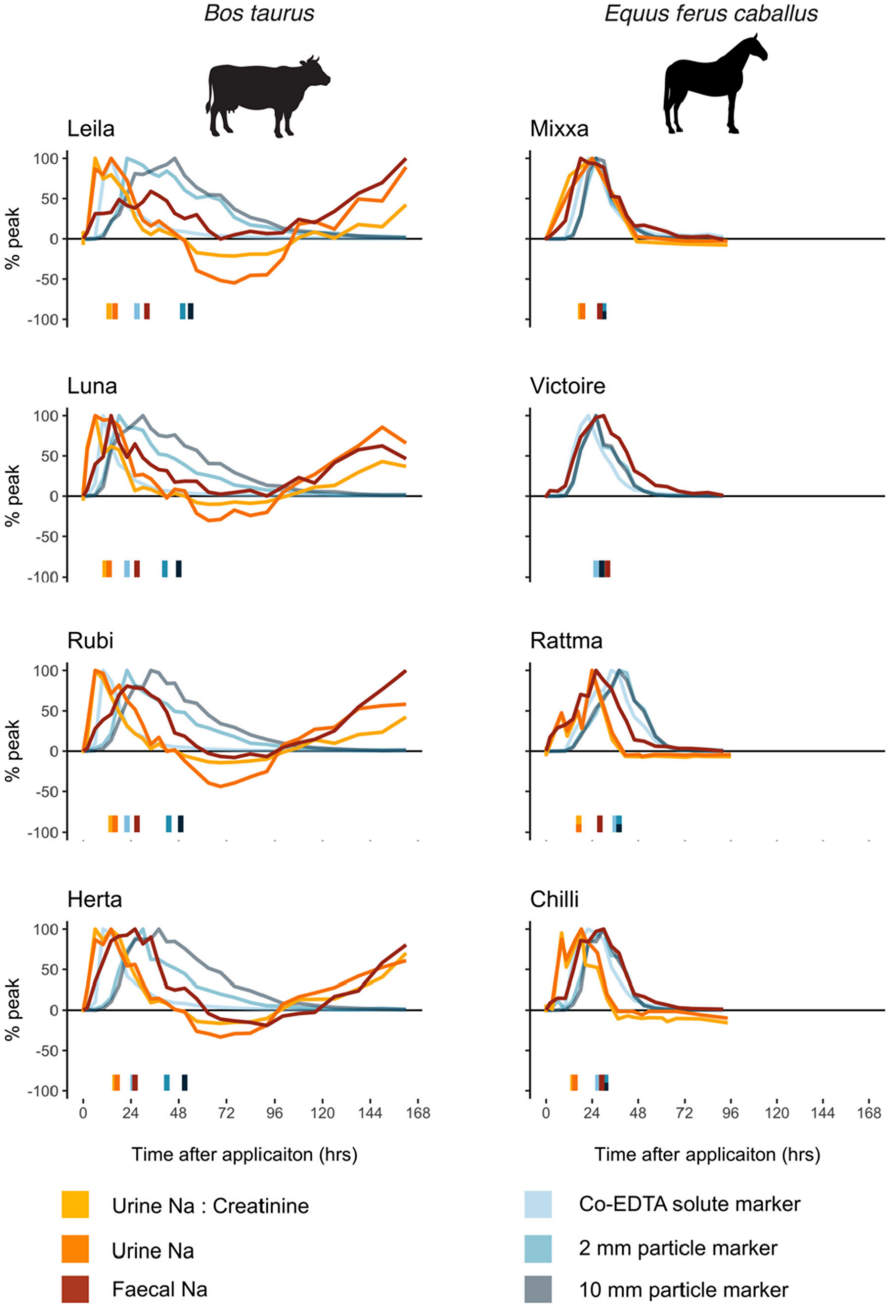
Excretion patterns and mean retention times of sodium (Na) and nonabsorbable marker substances following a pulse dose at t = 0 h. Observations were recorded over 7 days for cows (L) and 4 days for horses (R). All measurements have been standardized to baseline samples collected on the day before treatment (0%) and maximum concentrations observed during the experiment (100%). Negative values indicate concentrations below the baseline. Mean retention time (MRT) was calculated according to Thielemans et al. (1978) and are indicated for markers separately at the bottom of each graph. *Note:* urine markers were not calculated for one horse (Victorie) as not enough samples were collected from this individual.

**FIGURE 2:**
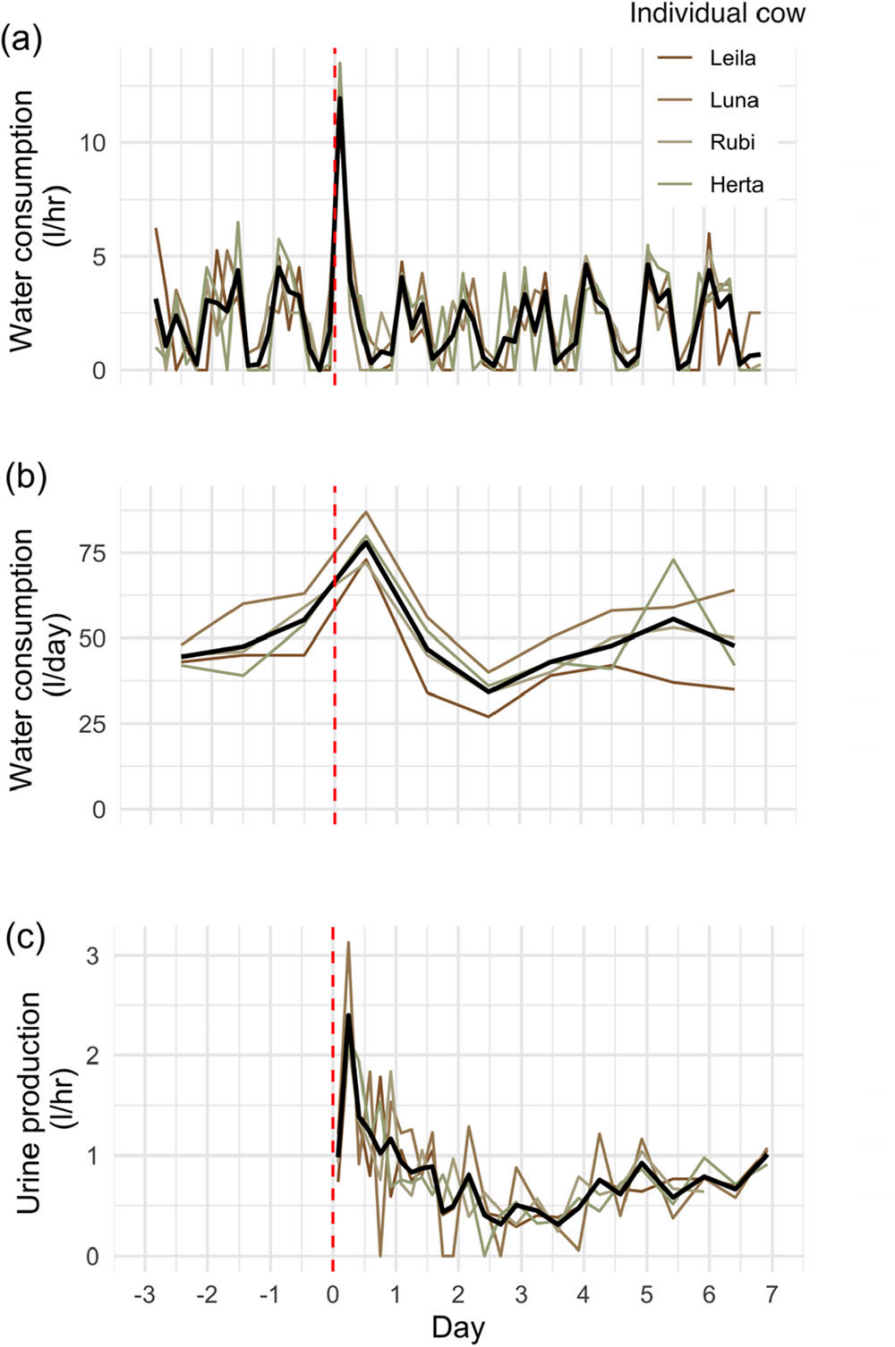
Water consumption and urine production by cattle (*Bos taurus*) during the sodium (Na) passage time experiment. The vertical dashed red line represents the time of Na adminstration; the thick black line represents mean water consumption or urine production across individuals. Water consumption displayed at 4‐h (a) and daily (b) intervals; urine production measurements were collected over progressively longer intervals (c). *Note:* urine production was not measured before the experiment starting to limit the period over which urinary canisters were attached to animals.

The mean food intake in cattle was 16.3 (SD = 2.0) kg dry matter per animal per day, representing a daily background Na intake of 84.5 ± 10.4 g per animal. Analogous results were not available for horses, although almost certainly lower as dietary Na concentrations were ~25 times lower. Following administration of the salt dose, Na excretion displayed a large peak in both urine and feces across individuals and species (Figure 1). In general, the MRT of excreted urinary Na (cattle: 15.5 ± 1.4 h; horses: 17.1 ± 1.6 h) was approximately half of that found for fecal Na (cattle: 28.0 ± 2.6 h; horses 29.5 ± 2.0 h; Table 1). Urine Na:Creatinine MRT was also slightly faster than urine Na MRT, indicating that immediately after salt dose, water intake, and hence urine volume and urinary Na dilution, also increased. This was confirmed by fine‐scale measurements of water intake and urine production in cows (Figure 2). Across individuals, the urinary MRT of Na:Creatinine was 49% and 56% of fecal Na MRT for cattle and horses, respectively (Table 1). Drooling was not observed in either cattle or horses, indicating that urine and feces were the major pathways of Na release.

Curiously, for cattle, where we provided a higher Na dose and measured over a longer period of time, we observed a second Na peak after > 96 h in both feces and urine (Figure 1). Simultaneously, these individuals consumed more water and produced higher volumes of urine during this second peak (Figure 2), and again were not observed drooling. We did not capture this effect for horses, where we provided a lower Na dose and only sampled for 96 h.

### 3.2 Sodium Recycling and Redistribution by Large Herbivores

**FIGURE 3:**
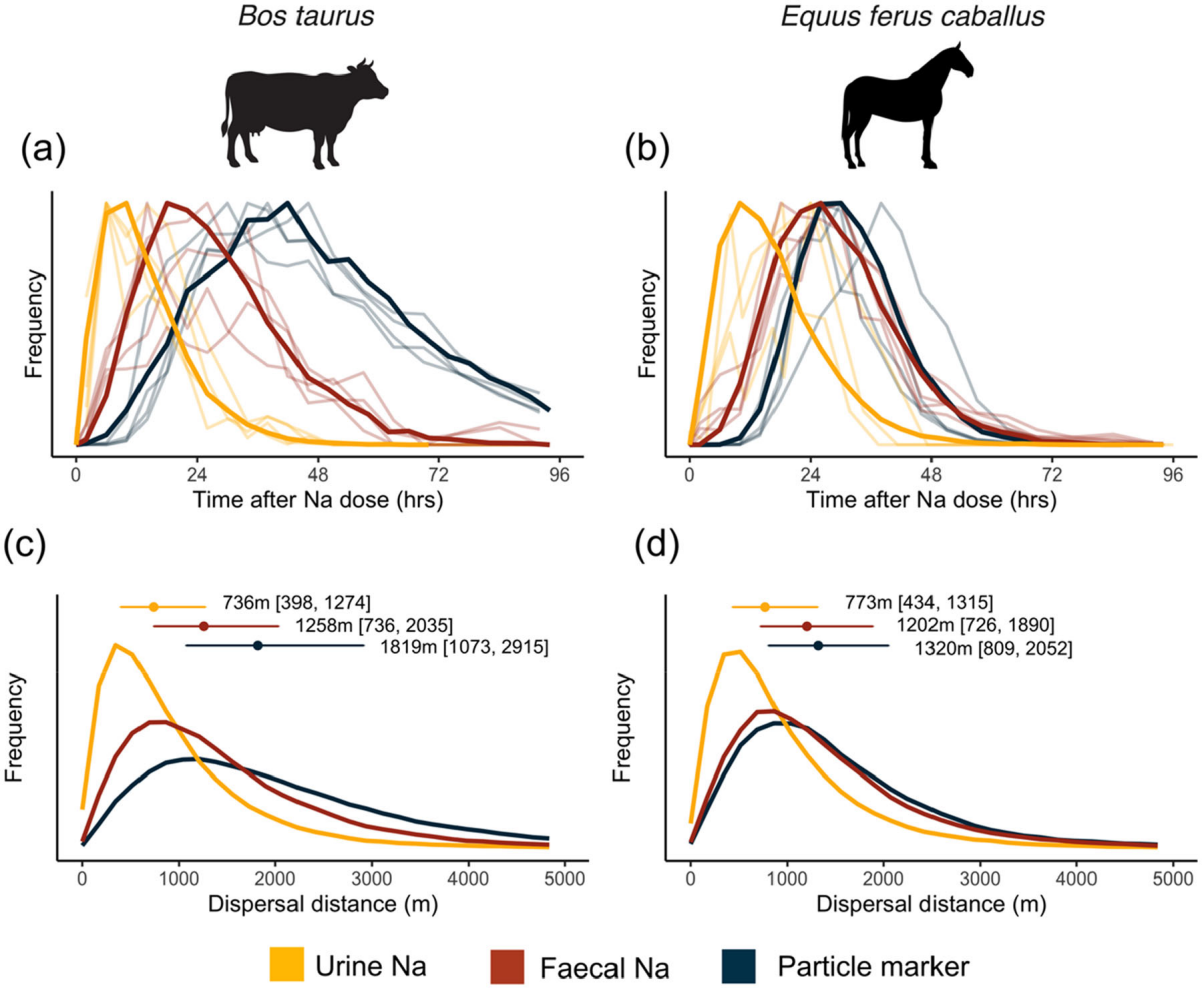
Comparison of sodium (Na) excretion curves via urinary and fecal pathways to the nonabsorbable 10 mm particle marker for cattle (a) and horses (b). Particle markers have previously been used to characterize the transit of nutrients—such as Na—through animal bodies. An average excretion curve for each pathway (solid lines) was generated by fitting a gamma distribution across individual animals. The mean and variance parameters used to generate each gamma distribution were then applied to a propagule dispersal model developed by Pires et al. (2018) to quantify variation in lateral sodium dispersal by cattle (c) and horses (d). respectively. All other values in the model were kept constant between simulations. The median dispersal distance and interquartile range for each of the urinary and fecal pathways are displayed above the density kernels. For cows, the median distance of urine dispersal was 41% and 60% shorter than fecal and particle dispersal, respectively. For horses, these values were 36% and 41%.

We found that incorporating differences in Na retention of different pathways had significant influence on lateral nutrient dispersal by large‐herbivores. For cattle, the shorter fecal and urinary retention times (Figure 3a) reduced estimated median Na transport distances by 31% and 60%, respectively, compared to the longer passage time of particle markers (Figure 3c). In horses, fecal and particle retention times were similar (Figure 3b) resulting in comparable dispersal kernels (Figure 3d). However, the urinary pathway was shorter, and estimated Na dispersal distances via this pathway were 36% and 41% shorter, respectively.

## 4 Discussion

**FIGURE 4:**
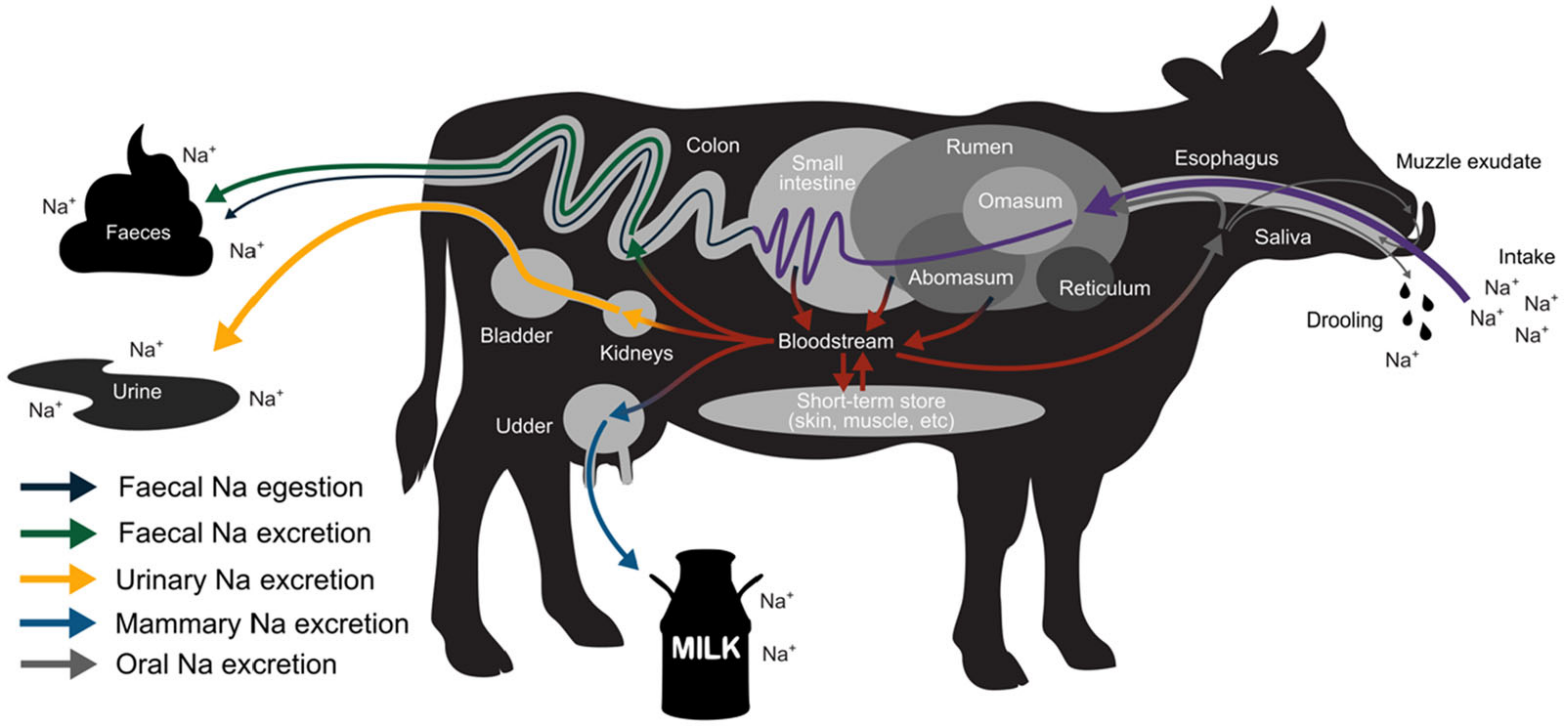
Schematic diagram of how sodium (Na) travels through the body of a cow (*Bos taurus*). Ingested Na is mostly (> 90%) absorbed into the blood stream in the upper gastrointestinal tract (rumen – small intestine), the small remainder travels through the digestive system and is egested as **fecal Na egestion**. The kidneys regulate the status of Na in the bloodstream by removing excess Na to the bladder for **urinary Na excretion**. Na is also additionally resecreted from the bloodsteam into the colon for **fecal Na excretion**. Notably, this means that Na can “overtake” the digesta, that is, a Na atom might be ingested with a certain food but be excreted via feces in the residue of food that was ingested earlier. Under certain conditions (e.g., during lactation), additional excretion pathways may be present, including **mammary Na excretion** in the form of milk. Minimal Na loss may also occur from **oral Na excretion** (e.g., drooling of saliva or muzzle exudate). The width of arrows indicates the relative contribution of each pathway to total Na release under normal conditions. When Na ingestion is high and the kidneys cannot regulate Na quickly enough, Na may also be moved into short‐term storage within body tissues (e.g., skin, muscle) for later release back into the bloodstream.

We examined the retention time of Na via different pathways through the body of large herbivores following administration of a large pulse dose of salt (NaCl). In general, we found that Na was released quicker via the urinary system compared to the fecal system, with mean Na retention times in urine approximately half that in feces (Table 1; Figure 1). Yet, both pathways for Na excretion (via urine and feces) were quicker than indigestible particles in feces (egestion; the typical method for estimating digesta retention time). This is because Na does not travel through the gastrointestinal tract like the non‐absorbed passage markers, but instead is almost completely absorbed (e.g., in cotransport with short‐chain fatty acids; [Stumpff 2018]) and then regulated by the kidneys for excretion in urine or resecreted into the large intestine for excretion in feces (Goff 2018). Notably, this means that Na can “overtake” the digesta it was consumed with—that is, a Na atom might be ingested with a certain food, but be excreted via feces in the residue of food that was ingested earlier. Figure 4 illustrates this “overtaking” mechanism, whereby Na absorbed into the bloodstream avoids slow passage through the entire gastrointestinal tract and can be excreted rapidly from the body. Such temporal differences have important implications for our understanding of animal Na physiology and subsequent impacts on ecosystems (e.g., via zoogeochemical processes).

### 4.1 Sodium Retention and Physiological Consequences

Comparison of the MRT and excretion pattern of Na in urine, feces and the nonabsorbable markers revealed insights into large herbivore Na physiology. The egestion patterns of the nonabsorbable fluid, small and large particle gut passage markers observed in our study confirm previously reported results for cattle and horses (Grandl et al. 2018; Hummel et al. 2018; Schwarm et al. 2022; Zhang et al. 2023b). In cattle, there was a distinct spacing of the three markers, whereby the longer retention of larger particle markers and shorter retention of the fluid marker corresponds to the forestomach “sorting” and “washing” mechanisms, respectively (Clauss et al. 2023). In horses, the three markers were excreted relatively simultaneously, in accordance with an absence of particle sorting or distinct digesta washing in this species (Hummel et al. 2018; Schwarm et al. 2022). The shape of the marker egestion curves (Figure 1) correspond to those proposed by reactor theory, where different types of reactors (aka “digestive tract morphologies”) determine the shape of the marker outflow from these reactors (Jumars 2000). This includes a rapid increase and long descending tail in cattle, whose digestive tract is dominated by the large forestomach (interpreted as a large, voluminous continuously stirred tank reactor, or, CSTR) and an equal increase and decrease in horses, in which the large intestine is interpreted as a series of many small CSTRs.

In contrast to differences in nonabsorbable marker MRTs, fecal and urinary Na MRT were similar in cattle and horses (Table 1). Importantly, we found that in both species, fecal Na did not closely follow the shape of passage trajectories for either the solute or particle markers (Figure 1). In cattle, fecal Na MRT was consistently longer than the solute passage marker (Co‐ETDA), but shorter than particle passage markers (2 mm and 10 mm). In horses, fecal Na MRT was shorter than passage markers for three individuals (Mixxa, Rattma and Chilli), but longer in one individual (Victoire) (Table 1). This demonstrates that most Na does not travel through the digestive tract of an individual animal in parallel with nonabsorbable markers, even if species averages of the different MRTs may subsequently appear similar. This is supported by the shape of the Na excretion curves, which partly deviate from those of the nonabsorbable markers: for urinary Na, there is a steeper increase and a longer decrease, corresponding to passage through the bladder as one large CSTR, whereas this shape is not typical for indigestible marker excretion in horses. For fecal Na, the increase is more moderate, even in cattle, corresponding to a release of Na into the large intestine (and its presumed series of small CSTRs [Figure 1]).

Our results thus demonstrate clear temporal differences between the renal and digestive systems for regulating excess Na from the body of large herbivores. Specifically, we show that following the intake of a large pulse of Na:

i. Increased water consumption is rapidly triggered (< 1 h) to dilute high Na concentrations (Bernal et al. 2023; Jansson and Dahlborn 1999). Specifically, we observed that cattle drank ~3 times more water in the 4 h after salt was administered than occurred at the same time period on other days (Figure 2a,b).
ii. Upon absorption of Na into the bloodstream, the kidneys quickly (< 20 h) removed some of the excess Na and excreted this via urine (Figure 1). Due to increased water consumption, urine production was also subsequently ~3 times higher during the 12 h after salt administration (Figure 2c); Na:Creatinine ratios revealed that this effect slightly lengthened our calculation of urinary Na MRT by ~1–2 h (Table 1).
iii. Feces represented a slower mechanism of Na excretion, with peak fecal Na concentrations occurring ~30 h after the pulse dose was provided in both cattle and horses (Table 1). When an animal is in Na deficit, Na is usually conserved from the large intestine (in exchange for potassium; K), but in situations of Na overload, the process can be reversed and Na actively secreted into the colon for excretion in feces (Argenzio et al. 1975; Argenzio and Stevens 1975). After ~48 h (water consumption and urine Na excretion) and ~72 h (fecal Na excretion) baseline levels were reached again in all animals.

It is likely that the magnitude and timing of Na retention and passage were influenced by the large amount of Na that was administered. Accordingly, our results may show an elevated response as the animals attempted to quickly rebalance Na homeostasis (Bernal et al. 2023). However, we believe our results may be broadly reflective of a general mechanism. For example, we observed similar results for cattle and horses regarding the magnitude and shape of Na retention times. Yet, these species ingested different initial salt dosages and were on markedly different Na diets (i.e., 5185 mg kg^‐1^ for cattle and 196 mg kg^‐1^ for horses). Furthermore, our results closely aligns with Lopes et al. (Lopes et al. 2004), who found a fecal Na peak ~30 h after sodium sulfate administration into the stomachs of seven horses.

### 4.2 The Curious Case of a Second Sodium Peak

For cattle, where we sampled for a longer period of time, we observed an unexpected second Na excretion peak in both urine and feces. The initial Na peak (0–72 h) was followed by a nadir where Na excretion was less than baseline samples (72–96 h) and finally a second peak (> 96 h). This pattern was remarkably consistent across fecal and urinary pathways and for all individuals (Figure 1). It was also mirrored by similar patterns of increased water consumption and urination rate after > 96 h (Figure 2). At the termination of our experiment (7 days) fecal and urine Na concentrations were similarly high as at the maximum of the initial peak, long after the passage markers had been entirely egested (Figure 1).

We believe that the observed second‐peak of Na excretion may be due to short‐term storage of Na in the body: a potential mechanism to quickly remove Na from bloodstream when in excess. Indeed, while kidneys are highly efficient at removing excess Na, bodily sodium balance is not just a renal affair (Titze 2014). Under conditions of high Na intake, large amounts of Na can be stored in the skin and muscle of animals without concomitant storage of water (Hofmeister et al. 2015) (Figure 4). This mechanism helps to quickly regulate blood Na concentrations and prevent hypertension (Bagordo et al. 2024). During the storage phase, active transport sequesters Na in bodily tissues before it is later released back into the blood for excretion via urine and feces once the animal's Na status is under control. Such effects have been observed before in rodents (e.g., Titze et al. 2003, 2005) and humans (e.g., Kopp et al. 2012); however, the exact mechanism of Na storage has yet to be elucidated. To our knowledge, our results present the first suggestion of similar extrarenal Na regulation for large herbivorous mammals and that such a mechanism even led to a temporary overcompensation by depleting Na below baseline levels (Figure 1).

The inference that mammals may temporarily store Na in body reservoirs has important implications considering large mammalian herbivores are disproportionately vulnerable to Na deficits (Duvall et al. 2023). Can the same mechanism observed to buffer an oversupplementation of Na also act to store Na when it is limiting? In many Na‐poor environments, large herbivores are observed consuming natural Na‐rich supplements (e.g., mineral licks [Duvall et al. 2023]), and such physiological adaptations for Na retention may compliment these behavioral responses. Additionally, in many domesticated or managed landscapes, considerable provisioning of artificial salt licks may emulate the high‐dose ingestion of Na described here. For example, large herbivores have been observed using artificial salt licks for > 1 h in the Kalahari Desert, a relatively Na‐poor environment (Abraham et al. 2023). The capacity of large mammalian herbivores to regulate Na in extrarenal tissues may be an important new area of research for large animal Na physiology and ecology.

### 4.3 Zoogeochemical Consequences of Different Sodium Transit Times

Due to increasing recognition of animal's important roles in elemental cycling (e.g., zoogeochemical processes), ecologists are increasingly interested in modeling animal‐mediated effects on biogeochemical cycles at broader scales (Abraham et al. 2023; Doughty et al. 2016; Schmitz et al. 2018). Such research critically relies on accurate estimates of nutrient consumption, retention time, and release in different forms (Abraham et al. 2021). For example, Na deposited in urine may alter rates of organic matter decomposition (Clay et al. 2015; Kaspari et al. 2009), whereas Na deposited in feces may be crucial for survivorship and fecundity of coprophagus insects (e.g., dung beetles; Pedersen and Zachariassen 2002). Previous studies have characterized the transit of nutrients through animal bodies by typically using fecal passage time markers. However, as we demonstrate here, this assumption critically overlooks the multiple nutrient retention and release pathways that occur in large herbivores, and thus may not be suitable for parameterization of nutrient excretion for elements such as Na. Furthermore, we show that cattle (ruminant) and horses (hindgut fermenter) have significantly different particle marker (egestion) retention times due to differences in their digestive physiology (Table 1; Grandl et al. 2018; Hummel et al. 2018; Schwarm et al. 2022; Zhang et al. 2023b). Yet, Na travels through their bodies in both urine and feces (excretion) at remarkably similar rates (Figure 1; Table 1). Estimating Na dispersal based on particle MRT would therefore create a difference between horses and cattle that does not exist.

Total nutrient transport by animals occurs as the sum of all egestion and excretion pathways (Subalusky and Post 2019). For Na, the contribution of each pathway varies depending on the animal's Na status (Hellgren and Pitts 1997). On high‐Na diets, sodium is ingested in excess and primarily excreted via urine; in low‐Na environments, feces increasingly become the dominate pathway of Na loss due to imperfect absorption (fecal Na egestion) and obligatory metabolic losses (fecal Na excretion) (Hellgren and Pitts 1997). Consequently, the impact of our results for improving quantification of Na redistribution will vary across geographic Na availability gradients. For example, in our study deviations in retention times were largest between urine Na and particle markers, especially for ruminants (Table 1; Figure 1). Therefore, previous modeling efforts that were parameterized using particle marker retention times (e.g., Doughty et al. 2016) have likely overestimated Na redistribution by large herbivores in high‐Na environments, such as coastal landscapes. It is, however, possible that currently unaccounted long‐distance Na dispersal may occur if large herbivores have extrarenal storage of Na in body tissues (see *Discussion* above) or weekly/monthly (circaseptan) patterns of Na excretion (Titze 2014). Future zoogeochemical modeling studies should examine if such effects occur and whether paramerterisation of models using the egestion pathway is appropriate for additional nutrients (e.g., N, P etc.). Where possible, modeling efforts should explicitly resolve differences in egestion and excretion pathways.

### 4.4 Caveats and Future Research

While our results for cattle and horses were generally consistent, they represent only two (domesticated) species. Further information is needed to unravel species‐ and individual‐level characteristics that could modify Na physiology and excretion. For example, we observed markedly different urination rate between cattle and horses. Due to a higher glomerular filtration rate, cattle urinate more regularly, often > 10 times daily (Orr et al. 2012), and we almost always collected urine during 4‐h intervals (Figure 2a). In this case, Na in the bladder will be voided (and thus measured) shortly after it has been regulated by the kidneys. Horses on the other hand, tend to urinate less frequently and thus Na may stay in the bladder for longer before release (Jansson and Dahlborn 1999). Individual‐level characteristics may further modify these trends; in our study we observed that two horses (Rattma and Chilli) had a much higher urination rate (4.5–5.5 times daily) than the other two horses (Mixxa and Victoire; 1.0–1.5 times daily). We anecdotally suggest that this may have been due to a clear dominance hierarchy, as Mixxa and Victoire spent more time preoccupied by dominating or evading others. Incidentally, the horse (Victoire) that was observed urinating least frequently also had the longest excretion period for Na via feces (Figure 1); this could suggest that a lack of urinary excretion led to an increased use of the fecal excretion pathway. It is also possible that individuals of the same species on the same Na diet develop differences in the dominant Na excretion pathways. For example, individual sheep (*Ovis aries*) can be classified as either fecal or urinary excreters depending on their dominant pathway (Michell and Moss 1992). Such species‐ and individual‐level idiosyncracies may have an appreciable impact on an animal's Na physiology and subsequent zoogeochemical effects. For example, occasional, but larger excretion of nutrients or the repeated return to specific sites (e.g., to middens) may create nutrient hotspots, whereas more continuous expulsion may homogenize nutrients more evenly across landscapes (Kaspari 2020).

In our experiment, we provided a very large dosage of salt (e.g., 400 g NaCl for cattle) to maximize the opportunity of observing a signal in retention times across both urinary and fecal pathways. Repeating our experiment with varying sized initial dosages and dietary Na intake will reveal the importance of Na ingestion magnitude on retention times. Furthermore, our experiment was conducted during the winter at low ambient temperatures (−5°C to 10°C), where animals had limited (horses) or no (cattle) exercise. Mean Na retention time in feces and urine may alter under conditions that promote alternative losses of Na (e.g., sweating during warm conditions or exercise). Particularly, for horses where sweating is used as the primary thermoregulatory mechanism this effect could be significant (Lindinger and Waller 2021; Rose et al. 1980). Lactation or drooling may also represent additional pathways of Na excretion (Suttle 2010).

Quantifying these additional pathways is likely to be context‐dependent. For example, drooling was not observed in the present study, in contrast to a study in which salivation was artificially enhanced pharmacologically in cattle (Zhang et al. 2023a). Daily saliva production of cattle can reach magnitudes of 250 L (Jiang et al. 2019; Maekawa et al. 2002), with saliva typically containing Na at ~150 mmol/L under normal feeding conditions (Bailey 1961; Bailey and Balch 1961; Post 1965; Thiangtum et al. 2017). Consequently, conditions that promote high levels of drooling or muzzle exudate may constitute an important loss of Na in cattle. While an exact quantification of saliva to the overall Na balance is missing in our study, we suspect that losses from this pathway are likely to be relatively minor in comparison to excretion via urine, feces, and milk. Although Na levels in saliva and muzzle gland secretions reflect Na deficiency or surplus respectively (Singh and Rani 1999; Bailey and Balch 1961; Murphy and Gartner 1974; Thiangtum et al. 2011), most saliva is swallowed along with the ingesta or digesta and muzzle gland secretions are very likely to be licked off by the animal. As a result, the Na concentration of rumen fluid closely follows that of saliva (Bailey 1961) and experimentally preventing saliva from being swallowed can induce Na deficiency (Singh and Rani 1999). Further research is required to elicit the conditions (if any) that demonstrate whether natural drooling causes significant losses of Na.

With their ease of handling and management, cows (as used in this study) might represent attractive model organisms to explore such questions further, including undertaking parallel measurements of blood pressure and examination of Na concentrations within different organs. However, we note that the use of a fistula bypasses the animal's taste buds, which may trigger different physiological responses (Hill and Mistretta 1990; McCaughey 1998). We did not observe apparent extrarenal regulation of Na in horses (where the Na dose was lower and where we also did not sample feces and urine long enough to test this). Additional research should examine if this process is also found in large‐hindgut fermenters, especially as the largest terrestrial mammals (e.g., elephants, rhinos) play disproportionately important roles in elemental cycling (Wolf et al. 2013) and possess this form of digestive physiology (Müller et al. 2013).

Finally, while the results presented here demonstrate clear differences in transit time through different pathways in an animal's body for Na, this pattern is unfortunately not generalizable across nutrients. Animals require 25+ elements to survive and reproduce (Kaspari and Powers 2016). Where, when, and the rate of nutrient assimilation through the gastrointestinal tract, and subsequent excretion via different pathways will be unique for each element (Robbins 2012), and further modified by the stoichiometric ratio within which multiple elements are ingested together. For example, nitrogen (N) is an essential macronutrient for animals, important for protein synthesis and various metabolic processes, and also excreted variably in feces, urine, and other pathways (e.g., sweat). In browsing herbivores, nitrogen may bind to plant secondary compounds (e.g., tannins) in the diet, increasing the fraction lost in feces (Leslie et al. 2008). In contrast, mammals that undergo long periods of fasting (e.g., hibernating bears or migrating whales) have evolved the capacity to reabsorb nitrogen from urea to minimize nutrient losses (Hellgren 1998). Using the example of Na, our study demonstrates that quantifying differences in nutrient retention time associated with such idiosyncracies may be critical for better understanding animal physiology and their subsequent zoogeochemical impacts.

## 5 Conclusion

We examined how sodium—an element vital for the health of animals and functioning of ecosystems—travels through the bodies of large herbivores. We show that following a pulse dose of Na, rapid assimilation into the bloodstream in the upper gastrointestinal tract and regulation via the kidneys and lower digestive tract can quickly excrete excess Na. Importantly, the speed with which large herbivores can resecrete Na from the bloodstream into the colon means that Na can “overtake” the digesta that it was consumed with. Despite the speed of such a process, we also infer that large herbivores have the potential for short‐term extrarenal Na storage (e.g., skin, muscle), allowing animals to additionally regulate Na from the bloodstream and gradually rerelease it once Na homeostasis is recovered. In addition to having important implications for understanding large herbivore physiology, accounting for differences in Na retention time is essential for accurately quantifying animal roles in nutrient dispersal and biogeochemical cycling. Together, our results indicate that previous research may have underestimated the physiological capacity of large herbivores to regulate excess Na, but overestimated their contribution to long‐distance Na dispersal.

## Author Contributions

A.J.A. and M.C. conceived the ideas and designed the methodology; A.J.A. and M.C. collected the data; B.R. and S.O. undertook laboratory procedures; A.J.A., M.C., and E.l.R. analyzed the data; A.J.A. led the writing of the manuscript. All authors contributed critically to the drafts and gave final approval for publication.

## Conflicts of Interest

The authors declare no conflicts of interest.

## Data Availability

The data that support the findings of this study are openly available in Abraham et al. Sodium retention in large herbivores at https://figshare.com/s/84fe28be65e2434f12d6. The datasets generated and analyzed during the current study will be made available at a Figshare repository upon publication.
